# High all-cause mortality and increasing proportion of older adults with tuberculosis in Texas, 2008–2020

**DOI:** 10.1017/S0950268824000669

**Published:** 2024-05-13

**Authors:** Belinda A. Medrano, Miryoung Lee, Gretchen Gemeinhardt, Lana Yamba, Blanca I. Restrepo

**Affiliations:** 1 Department of Epidemiology, School of Public Health, University of Texas Health Science Center at Houston, Brownsville, TX, USA; 2 Department of Management, Policy and Community Health, School of Public Health, University of Texas Health Science Center at Houston, Houston, TX, USA; 3 Tuberculosis Elimination Division, Texas Department of Health and Human Services, Austin, TX, USA; 4 Population Health Program, Texas Biomedical Research Institute, San Antonio, TX, USA; 5 School of Medicine, South Texas Diabetes and Obesity Institute, University of Texas Rio Grande Valley, Edinburg, TX, USA

**Keywords:** cavitation, delays, diabetes, foreign, older adults, tuberculosis

## Abstract

Pulmonary tuberculosis (PTB) elimination efforts must consider the global growth of the ageing population. Here we used TB surveillance data from Texas, United States (2008–2020; total *n* = 10656) to identify unique characteristics and outcomes in older adults (OA, ≥65 years) with PTB, compared to young adults (YA, 18–39 years) or middle-aged adults (40–64 years). We found that the proportion of OA with PTB increased from 15% in 2008 to 24% in 2020 (trend *p* < 0.05). Diabetes was highly prevalent in OA (32%) but not associated with adverse outcomes. Death was 13-fold higher in OA compared to YA and was 7% at the time of diagnosis which suggests diagnostic delays. However, once TB was suspected, we found no differences in culture, smear, or nucleic acid detection of mycobacteria (although less lung cavitations) in OA. During treatment, OA had less drug-resistant TB, few adverse reactions and adhered with TB treatment. We recommend training healthcare workers to ‘think TB’ in OA, for prompt treatment initiation to diminish deaths. Furthermore, OA should be added as a priority group to the latent TB treatment guidelines by the World Health Organization, to prevent TB disease in this highly vulnerable group.

## Introduction

*Mycobacterium tuberculosis* (*Mtb*) can cause latent tuberculosis infection (LTBI) in those infected but not sick, or active tuberculosis (TB) disease [1]. After two decades of a 2% annual decline in TB cases, in 2021 we still had an estimated 10.6 million cases and 1.6 million deaths [2]. The World Health Organization’s (WHO) ‘End TB Strategy’ is aiming at reducing TB incidence by 80% and TB deaths by 90% by 2030, compared with 2015, but its goals will not be reached at the current pace [3]. TB elimination efforts must be reaccelerated by focusing on populations at higher risk of TB.

Older adults (OA), that is, those 65 years and older, are one such group that represents an increasing burden of TB and worse TB treatment outcomes [4, 5]. This age group has the highest prevalence of latent TB in the United States [6] and is prone to immune-suppressive conditions that predispose them to reactivation of latent TB or new TB infection [7]. Delayed diagnosis occurs more frequently in OA due to fewer typical TB symptoms, TB diagnostic challenges, and existing conditions that mask TB disease [7–9]. We have also shown that the epidemiological profile of OA is different from that of younger patients, with fewer social risk factors for TB that complicates their identification [10]. OA are also more likely to live in congregate settings, such as nursing homes, that increase their risk of TB transmission [4].

The global population aged 65 years and over is growing faster than other age groups [11], and for the first time in the United States, OA are expected to outnumber children under the age of 18 by 2034 [12]. With the incidence of TB already shifting towards older people in many parts of the world [13, 14], more attention needs to be directed to TB in OA. However, there are relatively few studies of TB in OA populations and none, that we found, compared OA to two younger adult age categories in a large study [15, 16]. To address this gap, we have begun to conduct prospective studies in older versus younger adults with TB in a Hispanic-predominant community on the US-Mexico border [10, 17–19]. We recently reported on a retrospective study with thousands of patients using TB surveillance data from Tamaulipas, Mexico where we found that older people diagnosed with TB had features of a less complicated TB, less drug resistance and better treatment adherence, and yet, were more likely to die of any cause during TB (AOR 3.9; 95%CI: 2.5, 5.3) [19]. Here, we conducted a similar retrospective study on the other side of the US-Mexico border to identify unique features of OA with pulmonary TB (PTB) under a different health system. Namely, we sought to identify unique sociodemographics and clinical features of older PTB patients in a developed country like the United States, when compared to younger adults, and identify risk factors that predict adverse PTB outcomes in this age group. Our findings reveal an increasing proportion of OA with PTB over the 13-year period of this study, and highlight the case for diagnostic delays in this age groups given the important proportion of deaths at the time of diagnosis, before treatment has begun.

## Methods

### Study population

**Figure 1.**
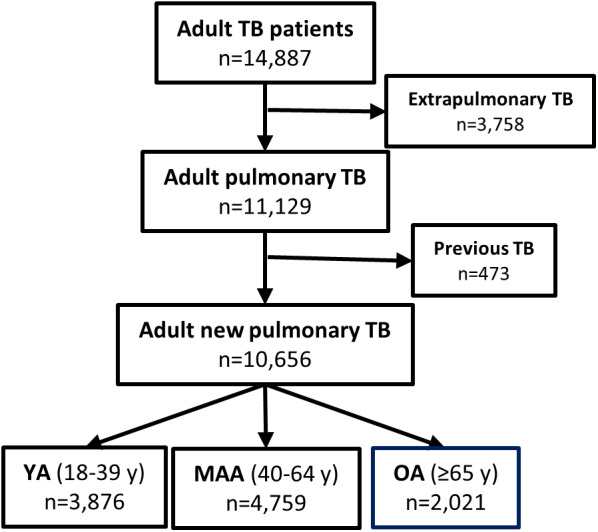
Flow chart of the study subject selection process. Patients with any extrapulmonary involvement (*n* = 3758) or previous TB (*n* = 473), were excluded for a final sample size of 10656. Pulmonary TB patients were divided into young adults (YA), middle-aged adults (MAA), and older adults (OA) for data analysis.

Analysis was performed using surveillance data created by the TB Elimination program from the Texas Department of State and Health Services (DSHS) between 2008 and 2020. There were 14887 adult TB patients reported in the state of Texas. Patients with extrapulmonary TB (*n* = 3758) and previous TB (*n* = 473) were excluded, leaving 10656 pulmonary TB patients for data analysis. TB patients were grouped into three age categories: young adults (YA; age 18–39 years; *n* = 3876), middle-aged adults (MAA; age 40–64 years; *n* = 4759), and OA (age 65 years and older; *n* = 2021) (Figure 1).

### TB case definitions

Confirmed TB cases met laboratory or clinical TB case criteria as defined by the Texas DSHS. Namely, laboratory diagnosis of TB included the isolation of *M. tuberculosis complex* by culture methods, its species identification using DNA probes or high-pressure liquid chromatography (HPLC), direct detection of *M. tuberculosis complex* from a clinical specimen by nucleic acid amplification tests (NAAT), or demonstration of acid-fast bacilli (AFB) when culture or NAAT results were not available. In the absence of laboratory confirmation, a clinical case of TB was met when patients had signs and symptoms compatible with active pulmonary TB disease, an abnormal chest radiograph or other chest imaging study, and a positive tuberculin skin test (TST) or interferon-gamma release assay (IGRA; T.Spot-TB, Oxford Immunotec or QuantiFERON versions not specified, Qiagen) for *M. tuberculosis*, plus current treatment with two or more anti-TB medications and a complete diagnostic evaluation. A clinical TB case included provider-diagnosed TB cases who improve on at least two anti-TB medications and cases identified at death based on autopsy or medical examiner reports.

### TB patient characteristics and treatment outcomes

Sociodemographics included age, sex, race or ethnicity, country of birth (United States, Mexico, or other), self-reported excess alcohol use, drug use (i.e., intravenous (IV) or non-IV), or being homeless in the past year. Residency in a correctional facility or long-term care facility was documented at the time of diagnosis. Comorbidities included diabetes (self-reported or laboratory-confirmed, but distinction not provided) and laboratory-confirmed human immunodeficiency virus (HIV) infection. TB characteristics at the time of diagnosis included the patient’s vital status (alive or dead) and abnormal chest X-ray results (including the presence of cavitations). Laboratory findings at diagnosis included results for AFB smears, *Mtb* cultures, NAAT, TST and/or IGRA. Drug resistance (DR) was available for first-line drugs except ethambutol, that is, isoniazid (INH), rifampin (RIF) and pyrazinamide (PZA), and 2nd line drugs when resistance was detected to first-line drugs. DR patterns included mono-resistance, multi-drug resistance (MDR; resistance to at least INH and RIF), pre-extensively drug resistance (Pre-XDR; resistance to INH, RIF and a fluoroquinolone, or resistant to INH, RIF and a second-line injectable (amikacin, capreomycin, and kanamycin)). Extensively drug-resistant TB (XDR-TB) was defined as resistance to INH, RIF, a fluoroquinolone and a second-line injectable, or resistant to INH, RIF, a fluoroquinolone, and bedaquiline or linezolid [20]. Additional DR patterns not categorized above were reported as ‘other drug resistance’. Patients were grouped into one of five PTB outcomes: Treatment completion, non-adherent to treatment either due to refusal to take treatment or lost to follow-up, treatment interruption due to an adverse event, moved/unknown, and death of any cause at the time of diagnosis or during TB treatment.

### Statistical analyses

Pearson’s chi-square test was used to compare categorical variables. Variables in bivariable analysis with *p*-values <0.20 were included as predictors in multivariable logistic regression with backward selection models, while retaining age and sex as key sociodemographics in final models. Abnormal chest X-ray was excluded from the multivariable analysis due to collinearity with cavitary disease. The following variables had a higher % of missingness and imputed with null entries: resident of a correctional facility (55.8%), diabetes (60.9%), and HIV (11.7%), given presumption that surveillance workers did not enter these data uniformly when patients did not have these characteristics. Results from imputed variables were in line with TB surveillance reports from Texas [21]. NAAT data was available for 48% of participants, so test results were only analyzed for the years 2018–2020 with data for 85% of the patients. About 59% of TST and IGRA results were missing and were deemed as non-random missing. Thus, these two results were not analyzed. Age was evaluated as an effect modifier (EM) of the associations between each predictor variable and TB outcomes (i.e., non-adherent or death of any cause) in simple logistic regression models. Significant interaction terms with *p*-values <0.05 were included in full multivariable models. Trends across age groups and across the study period, 2008–2020, were established by the score test for the trend of odds for categorical variables or the nonparametric test for trends across ordered groups, an extension of the Wilcoxon rank-sum test, for polytomous variables. Statistical significance was set at type I error (alpha) level < 0.05 for all tests. Data analysis was performed using STATA IC v.14 (Stata Corp LLC, College Station, TX).

## Results

### Characteristics of OA at the time of TB diagnosis

**Figure 2.**
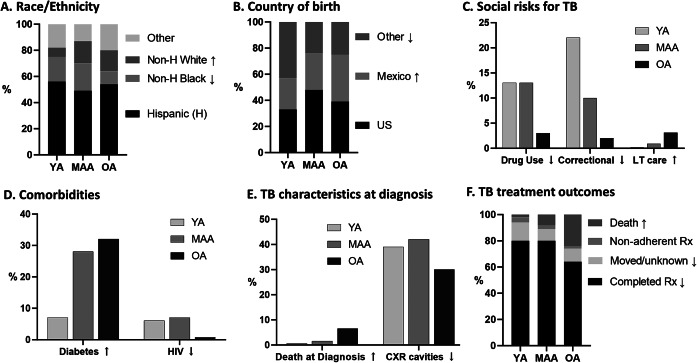
Significant trends with increasing age in characteristics of PTB patients. ↑ or ↓, increasing (↑) or decreasing (↓) trends across the YA, MAA, and OA age groups with trend *p* < 0.05. Correctional, resident of a correctional facility; H, Hispanic; LT care, resident of long-term care facility; MAA, middle-aged adults; OA, older adults; Rx, TB treatment; US, United States; YA, young adults.

Between 2008 and 2020 a total of 14887 TB cases were reported to the Texas DSHS. We selected those with new episodes of PTB (*n* = 10656; 75%) for data analysis (Figure 1). The final dataset consisted of 3876 YA, 4759 MAA and 2021 OA. Table 1 shows the characteristics of all adults, indicates significant differences between OA and the younger age groups, and shows p values for trends with increasing age. Figure 2 illustrates characteristics with significant trends across increasing age groups. Two-thirds of the patients were males, and this sex distribution did not change with older age. For race and ethnicity, the Hispanics comprised more than 50% across all age groups, but there was a decrease in non-Hispanic blacks from 19% in YA to 9.8% in OA, and an increase in non-Hispanic whites from 7.1% in YA to 16.1% in OA (Figure 2a). More than 50% of all adults were born outside of the United States, with a shift as age increased towards more Mexicans (24% in YA to 36% in OA) and fewer from other countries (44% in YA to 26% in OA; P trend <.001; Figure 2b). The OA group had the lowest proportions, as well as significant reductions, with older age in the following TB risk factors: excess alcohol use (10%), drug use (3%), homelessness (3%), and residence in a correctional facility (2%; Figure 2c for selected features). Residence in a long-term care facility increased with age (*P* for trend <.001; Figure 2c). For comorbidities, diabetes increased with age (32%; *P* trend <.001), while HIV decreased (0.7%; *P* trend <.001; Figure 2d). For TB-related characteristics at the time of TB diagnosis (Figure 2e), death from any cause was the highest in OA (7%; *P* trend <.001). Although the proportion of abnormal chest X-rays was similar across age groups, the detection of cavities decreased with age (*P* trend <.001). Detection of *Mtb* with AFB, cultures or NAAT was essentially similar across age groups. The use of NAAT increased over the study period (described below), with data between 2018 and 2020 suggested lower use in OA (from 86.6% in YA to 81.6% in OA). For TB outcomes, treatment completion decreased with age while deaths at diagnosis or during treatment increased with older age and was the main contributor to treatment interruption with increasing age (*P* trend <.001; Figure 2f). Whereas treatment outcomes were more detrimental in males among YA, there were no significant differences by sex among the OA group (Supplementary Table S1).

**Table 1.** Characteristics of Pulmonary TB patients by age group, Texas 2008–2020

	All adults	YA	MAA	OA	OA versus YA	OA versus MAA	Age trend
	(≥18 years)	(18–39 years)	(40–64 years)	(≥65 years)	*p*-value^a^	*p*-value^a^	*p*-value^b^ ^,^^c^
Total *n* (row %)	10656	3876 (36.4)	4759 (44.7)	2021 (19.0)			
Sociodemographics							
Age (mean, SD)	48 (18)	29 (6)	52 (7)	75 (7)			
Male	7224 (67.8)	2456 (63.4)	3450 (72.5)	1318 (65.2)	0.164	**<0.001**	**↑ 0.001**
Race/Ethnicity							
Hispanic	5568 (52.3)	2162 (55.8)	2309 (48.5)	1097 (54.3)	**<0.001**	**<0.001**	**↓ < 0.001**
Non–Hispanic Black	1919 (18.0)	737 (19.0)	984 (20.7)	198 (9.8)			
Non–Hispanic White	1389 (13.0)	276 (7.1)	788 (16.6)	325 (16.1)			
Other	1780 (16.7)	701 (18.1)	678 (14.2)	401 (19.8)			
Country of birth							
United States	4305 (40.4)	1262 (32.6)	2261 (47.5)	782 (38.7)	**<0.001**	**<0.001**	**↓ < 0.001**
Mexico	2996 (28.1)	922 (23.8)	1353 (28.4)	721 (35.7)			
Other	3355 (31.5)	1692 (43.7)	1145 (24.1)	518 (25.6)			
Risk factors for TB							
Excess alcohol use	1930 (18.1)	506 (13.1)	1225 (25.7)	199 (9.9)	**<0.001**	**<0.001**	0.314
Drug use	1176 (11.0)	489 (12.6)	636 (13.4)	51 (2.5)	**<0.001**	**<0.001**	**↓ < 0.001**
Homeless	568 (5.3)	111 (2.9)	406 (8.5)	51 (2.5)	0.448	**<0.001**	**↑ 0.021**
Correctional facility	1341 (12.6)	846 (21.8)	465 (9.8)	30 (1.5)	**<0.001**	**<0.001**	**↓ < 0.001**
Long–term care facility	114 (1.1)	8 (0.2)	44 (0.9)	62 (3.1)	**<0.001**	**<0.001**	**↑ < 0.001**
Comorbidities							
Diabetes	2284 (21.4)	285 (7.4)	1347 (28.3)	652 (32.3)	**<0.001**	**0.001**	**↑ < 0.001**
HIV	590 (5.5)	241 (6.2)	334 (7.0)	15 (0.7)	**<0.001**	**<0.001**	**↓ < 0.001**
TB–related characteristics							
Death at diagnosis	227 (2.1)	22 (0.6)	72 (1.5)	133 (6.6)	**<0.001**	**<0.001**	**↑ < 0.001**
Abnormal chest X–ray (*n* = 10216)	9766 (95.6)	3607 (95.8)	4355 (95.5)	1804 (95.5)	0.567	0.923	0.519
Cavities on chest X–ray (*n* = 9580)	3706 (38.7)	1373 (38.9)	1811 (42.3)	522 (29.6)	**<0.001**	**<0.001**	**↓ < 0.001**
Laboratory diagnostic tests							
AFB Smear + (*n* = 9876)	5681 (57.5)	2045 (55.7)	2661 (59.5)	975 (56.4)	0.630	**0.024**	0.168
*Mtb* Culture + (*n* = 9820)	7976 (81.2)	3038 (82.9)	3550 (79.9)	1388 (81.2)	0.133	0.263	**↓ 0.028**
NAAT TEST 2018–2020 (*n* = 1799)^d^	1799 (84.9)	634 (86.6)	765 (85.4)	400 (81.6)	0.153	0.165	**↓ 0.021**
NAAT + 2018–2020 (*n* = 1799)^d^	472 (26.2)	185 (29.2)	187 (24.4)	100 (25.0)	0.143	0.834	0.090
PTB outcome							
Completed treatment	8193 (76.9)	3107 (80.2)	3777 (79.4)	1309 (64.8)	**<0.001**	**<0.001**	**↓ < 0.001**
Moved/Unknown	1158 (10.9)	524 (13.5)	432 (9.1)	202 (10.0)			
Death at diagnosis or during Rx	923 (8.7)	78 (2.0)	371 (7.8)	474 (23.5)			
Non–adherent (Refused|Lost)	366 (3.4)	164 (4.2)	171 (3.6)	31 (1.5)			
Adverse event	16 (0.2)	3 (0.1)	8 (0.2)	5 (0.3)			

*Note:* Data expressed as *n* (column %) unless specified; *n* = 10656 unless *n* is shown; Bold values indicate statistically significant differences.

Abbreviations: AFB, acid-fast bacilli; IGRA, interferon-gamma release assay; MAA, middle-aged adults; *Mtb, Mycobacterium tuberculosis*; NAAT, nucleic acid amplification test; NHB, non-Hispanic Black; NHW, Non-Hispanic White; OA, older adults; Other, other race/ethnicity; TST, tuberculin skin test; YA, young adults.

a Chi-square test.

b Score test for trend of odds for categorical variables and the nonparametric test for trend across ordered groups, an extension of the Wilcoxon rank-sum test, for polytomous variables.

c Trend direction with respect to older age is indicated by arrows preceding the trend *p* values.

d NAAT testing and results were only evaluated between 2018 and 2020 when more than 80% of cases were tested.

### Resistance to TB drugs

Susceptibility testing results were available for 81% of all patients (Table 2). Resistance to any TB drug decreased with age (*P* trend = .008). There were no trends across age groups for mono-resistance to INH, RIF, PZA, or other drugs, but there was a decreasing trend in MDR-TB (*P* trend <.001), pre-XDR TB (*P* trend = .018), and XDR TB (*P* trend = .048) with older age.

**Table 2.** TB drug resistance prevalence by age group, Texas, United States, 2008–2020

		YA	MAA	OA	Age trend
	All adults	(18–39 years)	(40–64 years)	(≥65 years)	*p*-value^b^ ^,^^c^
Total *n*	10656	3876	4759	2021	
DR testing	8659 (81.3)	3153 (81.4)	3823 (80.3)	1683 (83.3)	0.200
DR^a^	1164 (13.4)	449 (14.2)	525 (13.7)	190 (11.3)	↓ 0.008
INH mono–R	310 (3.6)	126 (4.0)	130 (3.4)	54 (3.2)	0.124
RIF mono–R	6 (0.1)	2 (0.1)	4 (0.1)	0	0.582
PZA mono–R	99 (1.1)	35 (1.1)	38 (1.0)	26 (1.5)	0.278
MDR	87 (1.0)	43 (1.4)	40 (1.1)	4 (0.2)	↓ <0.001
Pre–XDR	12 (0.1)	8 (0.3)	4 (0.1)	0	↓ 0.018
XDR	3 (0.03)	3 (0.1)	0	0	↓ 0.048
Other DR	647 (7.5)	232 (7.4)	309 (8.1)	106 (6.3)	0.364

Abbreviations: DR, any TB drug resistance; INH mono-R, isoniazid monoresistance; MAA, middle-aged adults; MDR, multi-drug resistant; Pre-XDR, pre-extensively resistant; PZA mono-R, pyrazinamide monoresistance; OA, older adults; Other DR, drug resistance patterns not otherwise categorized; RIF mono-R, rifampin monoresistance; XDR, extensively drug-resistant; YA, young adults.

a Denominator for all drug resistance results is the n shown under DR testing.

b Score test for trend of odds for categorical variables.

c Trend direction with respect to older age is indicated by arrows preceding the trend *p* values.

### Age as a predictor of adverse outcomes

Among TB patients who did not die, nonadherence to TB treatment (refused or lost to follow-up) was less likely in OA compared to YA, although statistical significance was not reached (aOR 0.69, 95% CI 0.45, 1.05; Table 3). Instead, predictors of nonadherence to treatment in all age groups included male sex (aOR 1.70, 95% CI 1.23, 2.29), consuming excess alcohol (aOR 1.36, 95% CI 1.04, 1.78), being homeless (aOR 2.84, 95% CI 1.99, 4.06), residence in a correctional facility (aOR 4.45, 95% CI 3.42, 5.78) and being HIV positive (aOR 1.58, 95% CI 1.05, 2.37). Death from any cause at the time of diagnosis or during treatment, increased with old age and was 13.4 times higher for OA (aOR 13.44, 95% CI 10.12, 17.84) when compared to YA. Additionally, among all age groups, male sex (aOR 1.22, 95% CI 1.03, 1.45), residing in a long-term care facility (aOR 2.71, 95% CI 1.75, 4.19), and testing positive for HIV (aOR 2.40, 95% CI 1.78, 3.24) were associated with increased odds of death. Predictors protective against all-cause death included birth in Mexico (aOR 0.64, 95% CI 0.53, 0.77) or another foreign country (aOR 0.44, 95% CI 0.35, 0.54), when compared to the United States, residing in a correctional facility (aOR 0.24, 95% CI 0.14, 0.40), and having chest X-ray cavities (aOR 0.81, 95% CI 0.69, 0.96).

**Table 3.** Predictors of adverse TB treatment outcomes among TB patients of all age groups^a^

	Non-adherent^b^	Death^c^
Predictor variables	aOR (95% CI)	aOR (95% CI)
Age group		
YA (18–39 y)	1.00	1.00
MAA (40–64 y)	0.88 (0.69, 1.13)	**3.41 (2.58, 4.52)**
OA (≥65 y)	0.69 (0.45, 1.05)	**13.44 (10.12, 17.84)**
Male	**1.70 (1.23, 2.29)**	**1.22 (1.03, 1.45)**
Race		
Non–Hispanic White	1.00	
Non–Hispanic Black	**0.61 (0.42, 0.91)**	
Hispanic	0.80 (0.57, 1.10)	
Other	**0.63 (0.40, 0.99)**	
Country of birth		
United States		1.00
Mexico		**0.64 (0.53, 0.77)**
Other		**0.44 (0.35, 0.54)**
Alcohol use	**1.36 (1.04, 1.78)**	
Homeless	**2.84 (1.99, 4.06)**	
Correctional facility	**4.45 (3.42, 5.78)**	**0.24 (0.14, 0.40)**
Long–term care facility		**2.71 (1.75, 4.19)**
HIV	**1.58 (1.05, 2.37)**	**2.40 (1.78, 3.24)**
Cavities on chest X–ray		**0.81 (0.69, 0.96)**
AFB smear positive	**0.71 (0.57, 0.90)**	

*Note:* Bold values indicate statistically significant differences.

Abbreviations: AFB, acid-fast bacilli; aOR, adjusted odds ratio; CI, confidence interval; MAA, middle-aged adults; OA, older adults; YA, young adults.

a Predictor variables with *p* < 0.20 were included in the full regression models. All reduced models (shown) include age group and sex plus predictor variables with a *p* < 0.05.

b Non-adherent includes cases who did not die but refused treatment or were lost to follow-up when compared to those who completed. It excludes those who moved, unknown, or had an adverse event.

c Death from any cause at diagnosis or during TB treatment.

Among the OA group (Supplementary Table S2), homelessness was the only independent predictor of nonadherence to TB treatment (aOR 13.02, 95% CI 4.94, 34.33). The odds of death increased by 6% for each one-year increase in age and 131% for those with a positive *Mtb* culture (95% CI 1.55, 3.44). Birth in Mexico (aOR 0.74, 95% CI 0.56, 0.99) or another foreign country (aOR 0.48, 95% CI 0.33, 0.68) was protective for death when compared to OA born in the United States.

### Age as an effect modifier

We evaluated if a TB patient’s age would modify the association between different predictor variables and adverse TB treatment outcomes. In bivariate analysis, age was an effect modifier (EM) of the association between the adverse outcome, non-adherent, and the respective predictors: male sex (*P* value = .026) and born in Mexico (*P* value = .012) (Supplementary Table S3). These two EM variables were included in the full regression model for non-adherent, but were not significantly associated with treatment nonadherence and subsequently removed from the final model. Age did not modify the association between any of the host characteristics and death from any cause, as an outcome.

### Secular trends over the study period among the OA group

**Figure 3.**
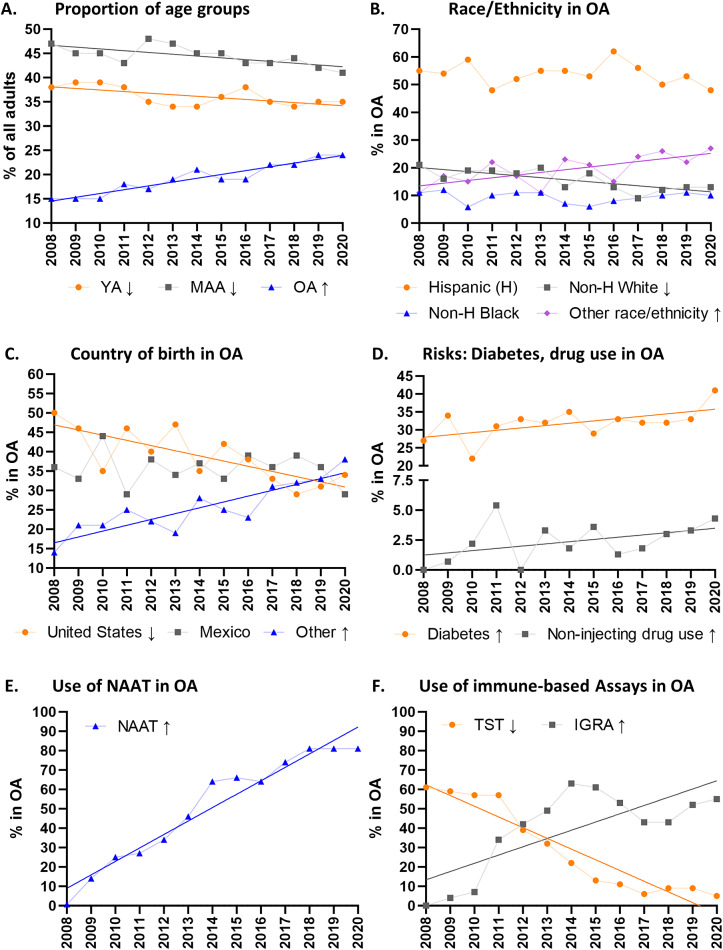
Significant trends between 2008 and 2020 in the proportion of age groups, the characteristics of older adults and methods used to support their TB diagnosis. Significant increasing (↑) or decreasing (↓) trends across age groups. Regression lines are shown for variables with significant trends. H, Hispanic; IGRA, IFN-gamma release assays; NAAT, nucleic acid amplification tests; MAA, middle-aged adults; Older adults (OA); TST, tuberculin skin test; YA, young adults.

We evaluated if there were changes in the prevalence and characteristics of OA with PTB over the study period, and how these may differ from changes in the YA or MAA groups. Table 4 shows trends for all age groups and Supplementary Table S4 for the OA group. Figure 3 illustrates significant findings for OA group: An increase in their proportion from 15% in 2008 to 24% in 2020 (Figure 3a); A lower proportion of non-Hispanic whites and higher individuals of other races/ethnicities (Figure 3b); Fewer US-born and more foreign-born from countries other than Mexico (Figure 3c); More non-injecting drug use and diabetes (Figure 3d); Less with abnormal chest X-rays and more with positive smears or cultures (Supplementary Table S4). The use of NAAT for *Mtb* detection increased over the study period, with more than 80% coverage in 2018–2020, and hence, these results were used for data analysis (Table 1 and Figure 3e for OA). There was increased use of IGRAs and reduction in TSTs over the study period in the OA group (Figure 3f). There were no reductions in the proportion of patients reported as dead at TB diagnosis or in adverse treatment outcomes during the study period.

**Table 4.** Trends across time (2008–2020) in characteristics of pulmonary TB patients, by age groups

	All Adults	YAA	MAA	OA
	(≥ 18 years)	(18–39 years)	(40–64 years)	(≥65 years)
	Trend *p* ^a^ ^,^^b^	Trend *p* ^a^ ^,^^b^	Trend *p* ^a^ ^,^^b^	Trend *p* ^a^ ^,^^b^
Proportion of each age group	Not apply	**↓ 0.003**	**↓ 0.003**	**↑ < 0.001**
Sociodemographics				
Male	0.881	0.588	0.654	0.395
Race/Ethnicity				
Hispanic	0.310	**↓0.007**	**↑ < 0.001**	0.468
Non–Hispanic Black	**↓ 0.044**	0.405	**↓ 0.016**	0.824
Non–Hispanic White	**↓ < 0.001**	**↓ 0.008**	**↓ < 0.001**	**↓ 0.008**
Other	**↑ < 0.001**	**↑ < 0.001**	**↑ < 0.001**	**↑ < 0.001**
Country of birth				
United States	**↓ < 0.001**	0.462	**↓ < 0.001**	**↓ < 0.001**
Mexico	0.138	**↓ < 0.001**	**↑ < 0.001**	0.693
Other	**↑ < 0.001**	**↑ < 0.001**	**↑ < 0.001**	**↑ < 0.001**
Excess alcohol use	**↓ < 0.001**	**↓ < 0.001**	**↓ < 0.001**	0.493
Drug use	0.079	0.175	0.569	**↑ 0.023**
IV drug use	**↓ 0.003**	0.063	**↓ 0.038**	0.344
Non–inject drug use	0.412	0.337	0.682	**↑ 0.044**
Homeless	0.362	0.153	0.169	0.992
Correctional facility	**↓ 0.005**	0.659	**↓ 0.016**	0.164
Long–term care facility	0.135	0.726	**↓ 0.019**	0.367
Comorbidities				
Diabetes	**↑ < 0.001**	0.524	**↑ < 0.001**	**↑ 0.030**
HIV	**↓ < 0.001**	**↓ 0.002**	0.238	0.605
TB–related characteristics				
Dead at TB diagnosis	0.931	0.639	0.827	0.114
Abnormal chest X–ray (*n* = 10216)	**↓ < 0.001**	**↓ < 0.001**	**↓ < 0.001**	**↓ < 0.001**
Chest X–ray cavities (*n* = 9580)	**↓ < 0.001**	**↓ < 0.001**	**↓ < 0.001**	0.242
AFB smear **+** (*n* = 9876)	0.184	0.568	0.210	**↑ 0.029**
*Mtb* culture + (*n* = 9820)	**↑ < 0.001**	0.388	**↑ < 0.001**	**↑ 0.011**
NAAT test performed	**↑ < 0.001**	**↑ < 0.001**	**↑ < 0.001**	**↑ < 0.001**
TST test performed	**↓ < 0.001**	**↓ < 0.001**	**↓ < 0.001**	**↓ < 0.001**
IGRA test performed	**↑ < 0.001**	**↑ < 0.001**	**↑ < 0.001**	**↑ < 0.001**
TB drug susceptibility testing	0.064	**↓ 0.014**	0.603	0.639
Drug–resistant TB (*n* = 8659)	0.664	0.681	0.496	0.262
Adverse outcomes				
Treatment not completed (*n* = 9020)	0.298	0.363	0.910	0.861
Death	0.124	0.325	0.750	0.665

*Note:* Total *n* is 10656 unless indicated; Treatment not completed includes failure to complete treatment due to any cause except death; Death refers to mortality of any cause at the time of diagnosis or during TB treatment; Bold values indicate statistically significant differences.

Abbreviations. AFB, acid-fast bacilli; IGRA, interferon-gamma release assay; MAA, middle-aged adults; MTB, *Mycobacterium tuberculosis* complex; NAAT, nucleic acid amplification; NHB, Non-Hispanic Black; NHW, Non-Hispanic White; OA, older adults; Other, Other Race/Ethnicity Not Specified; TST, tuberculin skin test; YA, young adults.

a Score test for trend of odds for categorical variables and the nonparametric test for trend across ordered groups, an extension of the Wilcoxon rank-sum test, for polytomous variables.

b Trend direction with respect to old age is indicated by arrows preceding the trend *p* values.

## Discussion

The proportion of OA diagnosed with PTB in the state of Texas increased significantly over the 13-year study period: from 15% in 2008 to 24% in 2020. Despite having more than 13-fold odds of death from any cause when compared to YA, OA had fewer social risks for TB, for example, less excess alcohol use, drug use, homelessness, HIV infection, and residence in a correctional facility. Diabetes occurred in more than one-third of OA but was not associated with adverse TB outcomes. This result is similar to our previous findings across all ages in Mexico [19, 22, 23], but contrasts with studies in adults where diabetes is a predictor of death [24]. While death during TB is known to be more prevalent in OA [25], a striking finding in our study was its 13-fold magnitude when compared to YA, as well as its reporting prior to TB diagnosis in nearly 7% of the cases, before TB treatment could be considered. Together, these findings indicate a smouldering challenge for TB control in Texas, and likely globally.

The high proportion of deaths at the time of TB diagnosis suggests delays in TB suspicion in OA, as shown for more than two decades [26]. There are several possible explanations for failure to consider TB in the differential diagnosis of OA. *First*, it has been suggested that older patients may have fewer ‘classical’ symptoms of TB [7, 26]. Our Texas dataset did not provide information on symptoms, but our prospective study in patients from the same Texas-Mexico region revealed that OA with TB were less likely to present with fever or chills (58% in OA vs. 81% in younger patients) [10]. *Second*, diagnostic delays may be due to lack of TB suspicion given the lower prevalence of known social risk factors for TB, as listed above, and as reported earlier [10, 19]. *Third*, even though abnormal chest X-rays were reported in over 95% of the TB patients, failure to consider TB in the differential diagnosis could be explained by the lower prevalence of cavitations in the OA group, which is a feature of active TB. The lower prevalence of cavitary TB in OA had also been reported previously [7, 27, 28], and may seem advantageous to the host because cavities hold a very large number of bacteria and are associated with poor treatment outcomes, prolonged culture conversion and higher Mtb transmission [29]. Cavities arise upon central necrosis of some lung granulomas, which are tissue nodules formed by the immune system to contain *Mtb* [30]. We posit that the lower prevalence of cavitary TB in OA reflects a declining immune response.

Age-related trends in race, ethnicity and country of birth can guide physicians to consider TB in the differential diagnosis of OA. In Texas, OA were predominantly Hispanic with most born in the US and closely followed by birth in Mexico. The reduced proportion of non-Hispanic blacks among OA may suggest death at a younger age in this race/ethnic group – this deserves further study. Regarding place of birth, the largest proportion of OA (39%) were born in the U.S., but over the study period, there was an increase in OA born in countries other than the U.S. or Mexico. A total of 124 countries of birth were represented in our study, including eight classified as high TB burden [2]. The shift from PTB patients born in the U.S. to other countries support the anticipated quadrupling of older immigrants in the U.S. by 2050 [31]. These changing demographics in Texas must be taken into consideration by TB elimination programs.

Once TB is considered among the differential diagnosis, the sensitivity of smears, cultures and NAATs was similar across age groups. The use of support methods for TB diagnosis shifted over the 12-year period, with increase in NAATs and IGRAs, and reduced TST. Between 2015 and 2020, more than half of OA patients had a NAAT test performed in Texas as part of their diagnostic workup, which is higher than the 2021 global average of 38% [32]. However, NAAT use was less prevalent in OA patients, and contrast with the WHO recommendation to promptly use rapid molecular test for quicker TB diagnosis in high-risk patients, such as OA [32]. The TST has poor sensitivity in OA due to immune defect in skin dendritic cells [33], while IGRAs are suitable for the detection of LTBI in OA [17]. Hence, the availability and overall performance of diagnostic tools for the detection of Mtb infection or disease in OA should not be a limitation for prompt TB diagnosis in Texas.

Treatment adherence is very high in Texas across all age groups given the strict enforcement of the Directly observed therapy (DOT) as the standard of care for TB [34], a contrast with the 7% abandon treatment and 2% treatment failure reported in the adjacent Mexican border [19]. In Texas, the trend for lower treatment completion with older age was due to the high prevalence of deaths at diagnosis or during treatment, but not to lack of adherence to treatment. On the adjacent Mexican border, OA adhered to TB treatment [19]. There were few adverse reactions in any age group, including OA, suggesting that TB treatment was well tolerated in this age group in our study population. However, this is not always the case. For example, in a meta-analysis, the odds of hepatoxicity in OA increased by 32% for the treatment of active TB, and by 414% for latent TB infection [35]. The authors recommended gentler treatment regimens for OA to minimize risks. We cannot rule out that the higher odds of death in OA in our study could be attributed in part to anti-TB treatment toxicity.

Despite treatment adherence, the odds of death was still higher in the OA group. Interestingly, in multivariable analysis of all age groups, foreign-born patients were less likely to die of any cause, suggesting an immigrant paradox [36]. We posit that non-US born TB patients are more likely to have had a previous exposure to Mtb that confers immunity that tapers TB severity [37, 38].

Strengths of this study include the large sample size that allowed adequate power to compare OA to young and middle-aged adults, and a span of 13 years to identify changes in the epidemiology of OA with TB in Texas. Limitations included the collection of data for TB surveillance with some information missing. Missing entries were imputed with null entries for resident of a correctional facility, diabetes, and HIV, which may underestimate the association of these risk factors with our outcome measures. Nevertheless, after imputation, the prevalence rates of these risk factors in our study were similar to those reported by the Texas DSHS TB program [39]. In contrast, imputation was not assumed to be valid for NAAT, TST, and IGRA testing given the not-at-random testing practices and changes in the frequency of their use over the study period. Hence, results from these tests were excluded from multivariable analyses. The surveillance dataset had limited information on the presence and duration of TB symptoms, to ascertain diagnostic delays or differential clinical presentation that could contribute to this problem. Finally, we cannot ascertain the relative contribution of TB versus other comorbidities to death, although this is a general limitation of studies on TB or in OA [25].

Together, our findings provide a foundation for recommendations. *First*, there is a need to educate physicians and public health workers to ‘think TB’ for prompt detection of the disease in OA. Once TB is considered in the differential diagnosis, *Mtb* detection is not compromised by old age, although less cavitary TB must be taken into consideration. Our *second* recommendation is to accelerate TB diagnosis- this could be lifesaving in OA. Clinicians should consider the WHO recommendation for simultaneous use of rapid molecular diagnostic tests and chest X-ray, rather than ordering molecular tests only after AFB smears are negative [40]. Once a TB diagnosis is established, TB treatment can begin. We found that OA were less likely to have DR-TB in Texas, and our results were similar across the Mexican border [19]. We also found that once treatment is initiated, OA in Texas and in Mexico are generally compliant, and few have adverse drug side effects. While higher deaths during TB treatment may be inevitable for OA given their higher fragility and multi-morbidities, we posit that this adverse event could be reduced by prompt diagnosis. Finally, we recommend the prioritization of OA in TB prevention efforts. Older adults are listed in the 2018 global targets for preventive TB treatment by the WHO, but not included among the high-risk groups for TB [2]. We propose their addition to the WHO’s TB infection management guidelines priority group for latent TB testing and preventative treatment [41]. This is feasible given that IGRA testing (but not TST) is suitable to identify TB infection in OA patients [17].

In summary, the growing proportion of OA with TB in Texas is likely to have international relevance, given the global growth of OA populations. The challenges we describe today for TB in OA, for example, delayed diagnosis and high death rates, have been noted for decades [42, 43]. Thus, the OA population requires attention given their higher risk of TB infection, latent TB reactivation, and death during TB.

## Supporting information

Medrano et al. supplementary material Medrano et al. supplementary material

## Data Availability

The datasets generated during and/or analyzed during the current study are available from the corresponding author in agreement with the Texas Department of State and Health Services on reasonable request and with approval from corresponding Internal Review Boards.

## Supplementary material

For supplementary material accompanying this paper visit http://doi.org/10.1017/S0950268824000669. click here to view supplementary material
