# Supplementary material for: High all-cause mortality and increasing proportion of older adults with tuberculosis in Texas, 2008–2020
Source: Epidemiol Infect. 2024 May 13;152:e82. doi: 10.1017/S0950268824000669 (PMC11131009; doi:10.1017/S0950268824000669)
Supplement: Medrano et al. supplementary material [file S0950268824000669sup001.docx]

| **Table S1**. Pulmonary TB treatment outcomes by sex among each age group | | | | | |  |  |  |  |  |  |
| --- | --- | --- | --- | --- | --- | --- | --- | --- | --- | --- | --- |
|  | **YA** | | |  | **MAA** | | |  | **OA** | | |
| **Treatment outcome** | **Male** | **Female** | **p-value^a^** |  | **Male** | **Female** | **p-value^a^** |  | **Male** | **Female** | **p-value^a^** |
| Completed Treatment | 1,882 (76.6) | 1,225 (86.3) | <0.001 |  | 2,682 (77.7) | 1,095 (83.7) | <0.001 |  | 848  (64.3) | 461  (65.6) | 0.123 |
| Moved/Unknown | 379 (15.4) | 145 (10.2) |  |  | 342  (9.9) | 90  (6.9) |  |  | 119  (9.0) | 83  (11.8) |  |
| Death at diagnosis or during Rx | 55  (2.2) | 23  (1.6) |  |  | 280  (8.1) | 91  (7.0) |  |  | 324  (24.6) | 150  (21.3) |  |
| Nonadherent (Lost \|Refused) | 139  (5.7) | 25  (1.8) |  |  | 142  (4.1) | 29  (2.2) |  |  | 23  (1.8) | 8  (1.1) |  |
| Adverse Event | 1  (0.0) | 2  (0.1) |  |  | 4  (0.1) | 4  (0.3) |  |  | 4  (0.3) | 1  (0.1) |  |

^a^ Chi-square test

**Table S2**. Predictors of adverse TB treatment outcomes among older adults ^a^

|  | **Non-adherent ^b^** | **Death^c^** |
| --- | --- | --- |
| **Predictor Variables** | **aOR (95% CI)** | **aOR (95% CI)** |
| Age^d^ | 1.02 (0.96, 1.07) | **1.06 (1.04, 1.08)** |
| Male | 1.26 (0.54, 2.92) | 1.16 (0.89, 1.53) |
| Country of Birth |  |  |
| United States |  | 1.00 |
| Mexico |  | **0.74 (0.56, 0.99)** |
| Other |  | **0.48 (0.33, 0.68)** |
| Homeless | **13.02 (4.94, 34.33)** |  |
| *Mtb* culture + |  | **2.31 (1.55, 3.44)** |

Abbreviations: aOR= adjusted odds ratio. CI, confidence interval; *Mtb, Mycobacterium tuberculosis*.

^a^ All models considered predictor variables with p <0.20 plus age group and sex, and reduced models are shown with predictor variables with p <0.05 plus age group and sex.

^b^ Treatment not completed excludes TB patients who died.

^c^ Death of any cause at diagnosis or during TB treatment

^d^ Age as continuous variable

**Table S3**. Evaluation of age as an effect modifier of the association between the predictor variables and adverse TB outcomes

|  | **Nonadherent** | **Death** |
| --- | --- | --- |
| **Predictor Variable** | **p-value**^b^ | **p-value**^b^ |
| Male | **0.026** | 0.785 |
| Race/Ethnicity |  |  |
| NHW | 1.00 | 1.00 |
| Other | 0.095 | 0.511 |
| NHB | 0.130 | 0.698 |
| Hispanic | 0.243 | 0.930 |
| Country of Birth |  |  |
| United States | 1.00 | 1.00 |
| Mexico | **0.012** | 0.577 |
| Other | 0.466 | 0.149 |
| Excess Alcohol Use | 0.640 | 0.656 |
| Drug Use | 0.877 | 0.947 |
| Homeless | 0.891 | 0.794 |
| Resident, Correctional Facility | 0.698 | 0.556 |
| Resident, Long-term Care Facility | 0.754 | 0.311 |
| Diabetes | 0.761 | 0.920 |
| HIV | 0.960 | 0.115 |
| Cavities on Chest X-ray | 0.212 | 0.321 |
| AFB Smear + | 0.742 | 0.528 |
| *Mtb* Culture + | 0.460 | 0.999 |
| Drug-resistant TB | 0.477 | 0.485 |

Note: NHW, Non-Hispanic White; Other, Other Race/Ethnicity Not Specified; NHB, Non-Hispanic Black; AFB, acid-fast bacilli; *Mtb, Mycobacterium tuberculosis*. P values ≤ 0.05 are in bold text.

a Nonadherent excludes TB patients who died.

b Death from any cause at diagnosis or during TB treatment
